# Does the novel artificial cervical joint complex resolve the conflict between stability and mobility after anterior cervical surgery? a finite element study

**DOI:** 10.3389/fbioe.2024.1400614

**Published:** 2024-06-03

**Authors:** Bing Meng, Xiong Zhao, Xin-Li Wang, Jian Wang, Chao Xu, Wei Lei

**Affiliations:** ^1^ Department of Orthopedics, Xijing Hospital, The Air Force Medical University, Xi’an, Shaanxi Province, China; ^2^ Department of Orthopedics, Affiliated Hospital of NCO School of Army Medical University, Shijiazhuang, Hebei Province, China; ^3^ Department of Knee Joint Surgery, Honghui Hospital, Xi’an Jiaotong University, Xi’an, Shaanxi, China; ^4^ Department of Health Statistics, Faculty of Preventive Medicine, the Air Force Military Medical University, Xi’an, Shaanxi, China

**Keywords:** artificial cervical joint complex, anterior cervical corpectomy and fusion, cervical disc arthroplasty, biomechanical, finite element analysis

## Abstract

**Background and objective:**

Our group has developed a novel artificial cervical joint complex (ACJC) as a motion preservation instrument for cervical corpectomy procedures. Through finite element analysis (FEA), this study aims to assess this prosthesis’s mobility and stability in the context of physiological reconstruction of the cervical spine.

**Materials and methods:**

A finite element (FE)model of the subaxial cervical spine (C3-C7) was established and validated. ACJC arthroplasty, anterior cervical corpectomy and fusion (ACCF), and two-level cervical disc arthroplasty (CDA) were performed at C4-C6. Range of motion (ROM), intervertebral disc pressure (IDP), facet joint stress (FJS), and maximum von Mises stress on the prosthesis and vertebrae during loading were compared.

**Results:**

Compared to the intact model, the ROM in all three surgical groups demonstrated a decline, with the ACCF group exhibiting the most significant mobility loss, and the highest compensatory motion in adjacent segments. ACJC and artificial cervical disc prosthesis (ACDP) well-preserved cervical mobility. In the ACCF model, IDP and FJS in adjacent segments increased notably, whereas the index segments experienced the most significant FJS elevation in the CDA model. The ROM, IDP, and FJS in both index and adjacent segments of the ACJC model were intermediate between the other two. Stress distribution of ACCF instruments and ACJC prosthesis during the loading process was more dispersed, resulting in less impact on the adjacent vertebrae than in the CDA model.

**Conclusion:**

The biomechanical properties of the novel ACJC were comparable to the ACCF in constructing postoperative stability and equally preserved physiological mobility of the cervical spine as CDA without much impact on adjacent segments and facet joints. Thus, the novel ACJC effectively balanced postoperative stability with cervical motion preservation.

## 1 Introduction

Cervical spondylotic myelopathy (CSM) is a degenerative disease caused by progressive spinal canal narrowing and chronic compression of the spinal cord (McCormick et al., 2020). This results in sensorimotor disturbances such as numbness of the limbs, muscle weakness, and unsteady gait, severely compromising the patient’s quality of life. When spinal cord compression occurs at the vertebral body level, including disc herniation extending over the disc level, severe osteophytes, and ossification of the posterior longitudinal ligament (OPLL), anterior cervical corpectomy and fusion (ACCF) is the conventional surgical choice, as it provides ample surgical exposure and allows for thorough decompression at the vertebral body level (Chen et al., 2016; Pescatori et al., 2020; Li et al., 2021). However, the fusion alters the kinematics of the cervical spine, leading to increased intradiscal pressure and excessive wear of the facet joints, ultimately progressing to adjacent segment degeneration (ASD) (Hua et al., 2020a; Rudisill et al., 2022; Toci et al., 2022). It has been reported that approximately 25%–92% of patients will exhibit radiographic changes at adjacent segments 10 years after the cervical fusion procedure, and 9%–17% of them eventually develop symptomatic disease that requires additional surgical intervention (Cho and Riew, 2013).

Artificial cervical disc prosthesis (ACDP) is the most developed, extensively applied, and well-researched motion preservation device (Latka et al., 2019), aiming to reduce the incidence of ASD by maintaining the natural kinematics of the cervical spine (Kim et al., 2023). However, cervical disc arthroplasty (CDA) is not a suitable substitute for ACCF due to different indications. Furthermore, this procedure potentially causes an abnormal excessive postoperative motion, which can compromise the stability of the cervical spine and may lead to heterotopic ossification (HO), posing a risk of a decrease in range of motion (ROM) at the index level (Wang et al., 2021). Approximately 32.5% of the post-CDA patients experienced HO, and 11.0% developed ROM-limited HO, which may eventually progress to secondary fusion (Hui et al., 2020). This outcome directly contradicted the primary purpose of ACDP, which aims to preserve the segmental motion.

To address the immobility issue associated with ACCF and the instability problem that comes with CDA, we have developed a novel artificial cervical joint complex (ACJC) grounded in the principle of spinal physiological reconstruction. The present study examines the biomechanical properties of three distinct types of anterior cervical surgeries above via finite element analysis (FEA) to evaluate whether the ACJC can balance postoperative stability with cervical motion preservation.

## 2 Methods and materials

### 2.1 Establishment of C3–C7 finite element models

The subaxial cervical spine (C3-C7) finite element (FE) model was constructed based on the computed tomography (CT) scanning images (LightSpeed 128-slice spiral CT, GE Healthcare, United States, 0.625-mm scanning slice thickness) of a consented volunteer (gender: male, age: 30, height: 170 cm, weight: 60 kg), without a history of cervical disorders, which was approved by the Medical Ethics Committee of Xijing Hospital (SN: KY20202040-F-1, 2020-10-29). The CT images were imported into Mimics 21.0 (Materialise Technologies, Leuven, Belgium) for conversion into a detailed C3-C7 facet model. Then, the geometric model underwent optimization in Geomagic Studio 2014 (Geomagic Inc., NC, United States), resulting in a refined non-uniform rational B-spline (NURBS) surface representation. Subsequently, the FE model was comprehensively prepared in Hypermesh 11.0 (Altair Engineering Corp., MI, United States), including the assignment of material properties, mesh generation, application of load and boundary conditions, and definition of component interactions. Finally, FEA was executed using MSC. Patran/Nastran 2012 (NASA Company, United States), ensuring a rigorous biomechanical simulation of the subaxial cervical spine.

The geometric model of the subaxial cervical spine comprised the vertebral bodies (cancellous and cortical bone), cartilaginous endplates, intervertebral discs (nucleus pulposus and annulus fibrosus), facet joints, and ligaments (including the anterior and posterior longitudinal ligaments (ALL and PLL), ligamentum flavum (LF), capsular ligament (CL), supraspinous ligament (SSL), and interspinous ligament (ISL)). The cortical bone and endplate thickness were defined as 0.5 mm. The composition ratio of nucleus pulposus (40%) to annulus fibrosus (60%), while the fiber layers were crisscross distributed and angled at a degree of 25° to the endplate (Lee et al., 2011; Hua et al., 2020b; Manickam et al., 2021). The interface of facet joints was simulated as surface-to-surface contact with a friction coefficient of zero, in alignment with previous studies (Yuchi et al., 2019; Cai et al., 2020), and the contact interfaces of the other cervical components were assigned to be completely bonded. The ligaments were considered unidirectionally tensioned one-dimensional non-linear spring materials, using 1D Spring elements to ensure the realistic transmission of forces during the FE simulation (Manickam et al., 2021; Sun et al., 2021). The mesh convergence test was performed to obtain an accurate FE model. The intact C3-C7 model contained 87,969 nodes and 275,980 elements, with an average mesh size of 0.15 mm (ranging from 0.05 mm to 0.5 mm). The aspect ratio of mesh elements was within 1:3, and the change rate of the maximal von Mises stress was within 5% (Dai et al., 2022), with no distorted meshes observed, met the convergence requirements.

According to the published literature (Lee et al., 2011; Yuchi et al., 2019; Hua et al., 2020b; Sun et al., 2021), the detailed material properties of the subaxial cervical spine components are described in Table 1.

**TABLE 1:** Material properties of subaxial cervical model components and surgery instruments.

Component	Young’s modulus (MPa)	Poisson’s ratio	Cross section area (mm^2^)
Cortical bone	12,000	0.29	—
Cancellous bone	450	0.29	—
Endplate	500	0.40	—
Annulus fibrosus	3.4	0.40	—
Nucleus pulposus	1.0	0.499	—
Facet joint cartilage	10	0.40	—
Anterior longitudinal ligaments (ALL)	30	0.3	6.0
Posterior longitudinal ligaments (PLL)	20	0.3	5.3
Capsular ligament (CL)	20	0.3	46.6
Ligamentum flavum (LF)	1.5	0.3	50.1
Interspinous ligament (ISL)	1.5	0.3	13.1
Supraspinous ligament (SSL)	1.5	0.3	5
Cobalt-chromium-molybdenum alloy (Co–Cr–Mo)	210,000	0.32	—
Titanium alloy (Ti6Al4V)	116,000	0.35	—
Ultra-high molecular weight polyethylene (UHMWPE)	3,000	0.49	—

### 2.2 FE model validation

To verify the structural integrity, material parameterization, and boundary condition constraints of the intact model, the present investigation secured all nodal points on the caudal surface of C7 and loaded axial compressive force upon the cranial surface of C3 (50 N, 1.0 N m), to mimic and assess the subaxial cervical spine’s biomechanical responses and kinematic competencies. The resultant ROM data for each segment under different loading directions were then cross-referenced with the findings from extant *in vitro* study (Panjabi et al., 2001; Liu et al., 2016) and the simulation research conducted under analogous conditions to affirm the validity of the FE model (Wu et al., 2019; Sun et al., 2020).

### 2.3 Design of ACJC prosthesis

Based on our preliminary research (Wu et al., 2012; Tan et al., 2015), the present study has developed an innovative ACJC prosthesis as a motion-preservation device that may be an alternative to ACCF surgery.

The ACJC prosthesis comprises two endplate components and an intermediate vertebral body component. These components are connected via upper and lower ball-and-socket joints. The endplate and vertebral body components are fabricated from titanium alloy (Ti6Al4V), the joint balls are constructed from cobalt-chromium-molybdenum (Co–Cr–Mo) alloy, and the socket parts are made of ultra-high molecular weight polyethylene (UHMWPE). Both endplate components are designed in an “L” shape, consisting of an anterior plate and a base. The anterior plate of the upper endplate component forms an angle less than 90° with its base, whereas the lower endplate forms an angle greater than 90° to conform to the anatomical shape of the human cervical vertebral body. The superior and inferior surfaces of the vertebral body component are inclined anteriorly to posteriorly in the sagittal plane, fitting the natural anterior-posterior slope of the human cervical spine. Two cavities are formed in the vertebral body component for installing the UHMWPE sockets. The joint ball is attached to the base of the endplate component and forms an internal joint with the socket capable of flexion, lateral bending, and axial rotation while completely restricting the translation in all directions. Although the translation is completely limited at a single joint, the complex kinematics of the cervical spine can be replicated through the coupled motion of the superior and inferior joints.

In the novel ACJC prosthesis, we have incorporated a new design of external joints, also a ball-and-socket joint structure, formed by the convex surfaces of the upper and lower endplate components and the concave surfaces of the vertebral body component. These external joints are in concert with the internal joints to participate in the prosthesis’s motion. It can limit the range of motion of the internal joint to 7° in various directions to avoid excessive motion and minimize the impact and wear between components. Additionally, the surface of the novel prosthesis that interfaces with the bone features a porous titanium structure with bone-like trabeculae. The porous titanium structure adopts a dodecahedral grid micro-pore type reported in the literature for its excellent osteogenic effects, with pore sizes ranging from 0.4 to 0.6 mm, grid wire diameters from 0.2 to 0.3 mm, porosity between 60% and 70%, and grid thickness at 1 mm (Huang et al., 2022; Bandyopadhyay et al., 2023). Both improvements above are intended to enhance the stability of the index segments while preserving the mobility inherent to the prosthesis.

**FIGURE 1:**
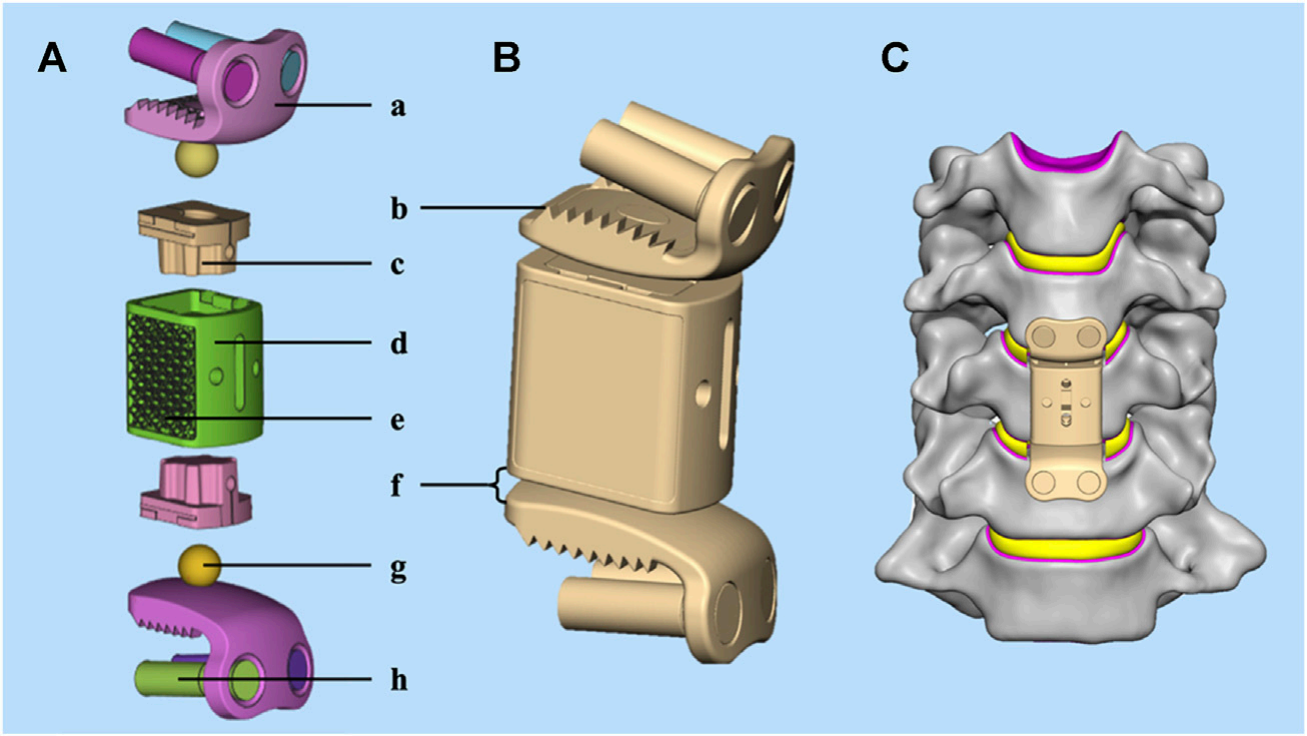
**(A)** The structure of the novel ACJC prosthesis: (a) the “L” shape endplate component; (b) the anti-pullout endplate teeth; (c) the UHMWPE joint socket; (d) the vertebral body component; (e) the porous titanium structure; (f) the external joint; (g) the Co–Cr–Mo alloy joint ball; (h) vertebral screws. **(B)** Three-dimensional geometric model; **(C)** the implanted view.

The ACJC prosthesis is fixed to the superior and inferior vertebral bodies following subtotal corpectomy via vertebral screws. Primary stability is achieved through the endplate teeth’s anti-pullout configuration and the screw fixation. Meanwhile, osteointegration is facilitated by new bone ingrowth into the prosthesis’s porous structure, establishing a robust titanium-bone interface that ensures enduring stability for the index segment (Figure 1).

### 2.4 Establishment of three types of anterior cervical surgery models

**FIGURE 2:**
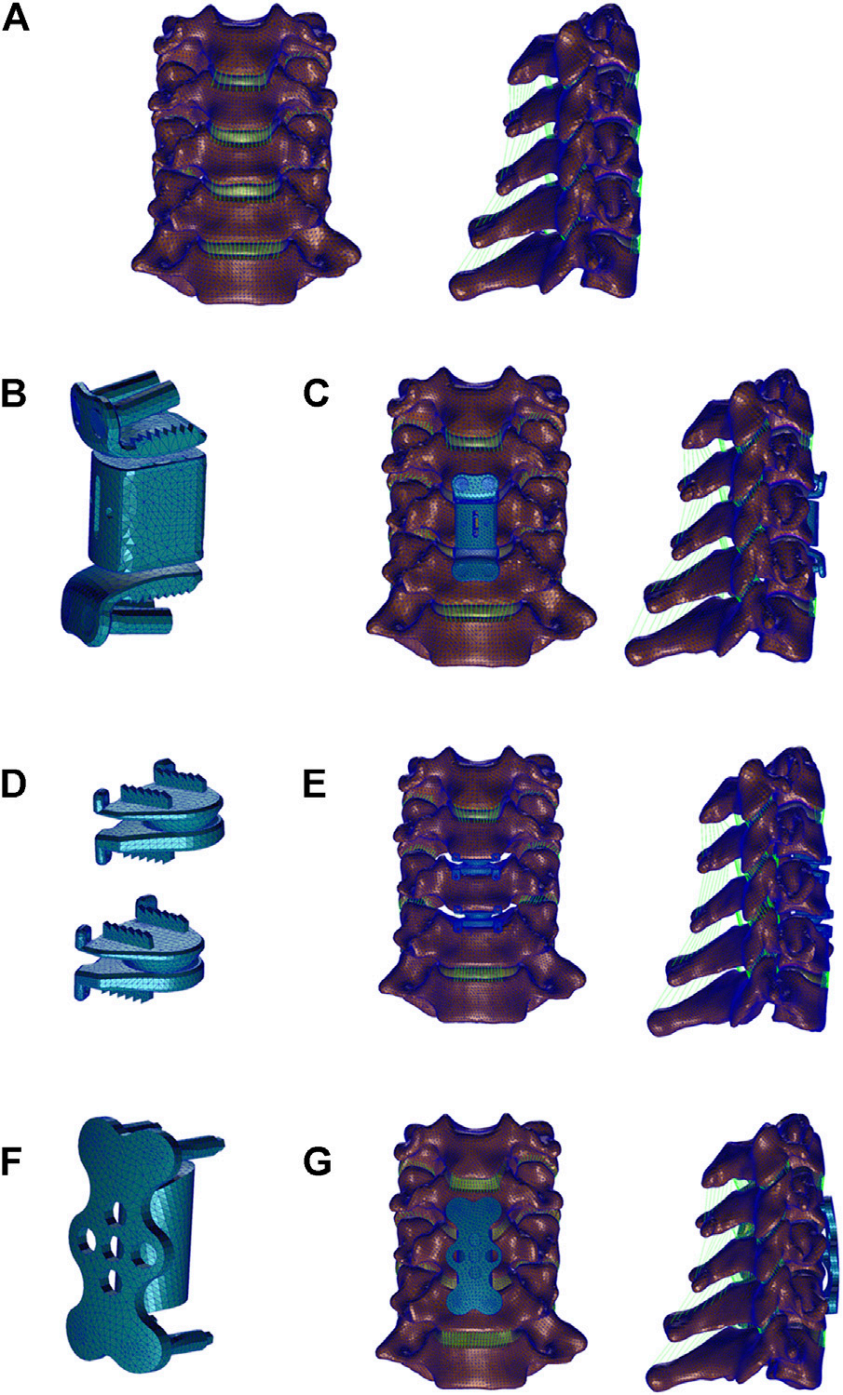
Three-dimensional finite element models of Intact subaxial cervical spine (C3-C7) and three anterior cervical surgery were established. **(A)** Front and lateral view of intact C3-C7 model; **(B)** The artificial cervical joint complex (ACJC) prosthesis; **(C)** ACJC implanted after C5 corpectomy; **(D)** Prestige-LP prostheses; **(E)** Two-level CDA at the C4-C6 level. **(F)** The titanium mesh cage (TMC) with plate and screws; **(G)** Anterior cervical corpectomy and fusion (ACCF) at the C4-C6 level.

All three surgical models were established in the validated C3-C7 model (Figure 2A).

The ACCF and ACJC arthroplasty were performed, respectively, following subtotal corpectomy at the C5 level. In the ACCF model (Figures 2F, G), a conventional titanium mesh cage (TMC) (Weigao, Shandong, China) packed with autogenous bone grafts was inserted into the post-corpectomy space, and the C4-C6 segments were secured by plate and screws (Weigao, Shandong, China). The ACJC arthroplasty technique (Figures 2B, C) parallels that of ACCF. The body component of ACJC was situated within the post-corpectomy cavity, and the superior and inferior endplate components were then affixed to the C4 and C6 vertebral bodies, respectively, using screws (Weigao, Shandong, China). In the two-level CDA model, the Prestige-LP prostheses (Medtronic Inc., MN, United States) were inserted into the post-discectomy spaces at C4-5 and C5-6 levels (Figures 2D, E). The intervertebral disc, ALL, and PLL at the index segments were removed. Following endplate removal, the contact area between the prostheses and the vertebral bodies was maximized. The vertebral screws were modeled as cylindrical shape, and the interfaces of prosthesis-screws and vertebra-screws were subjected to tie constraint and with no relative motion, considered complete fusion, according to reported literature (Hua et al., 2020a; Guo et al., 2021). The Co–Cr–Mo alloy-UHMWPE surface in the ACJC model and the Ti-Ti surface in the Prestige-LP model were set as a surface-to-surface contact with a coefficient of friction of 0.1 (Bhattacharya et al., 2011; Choi et al., 2021).

The ACCF surgical model included 48,174 nodes and 209,273 elements, the two-level CDA surgical model included 90,517 nodes and 368,813 elements, and the ACJC arthroplasty model included 77,618 nodes and 268,691 elements.

### 2.5 Loading and boundary condition

**FIGURE 3:**
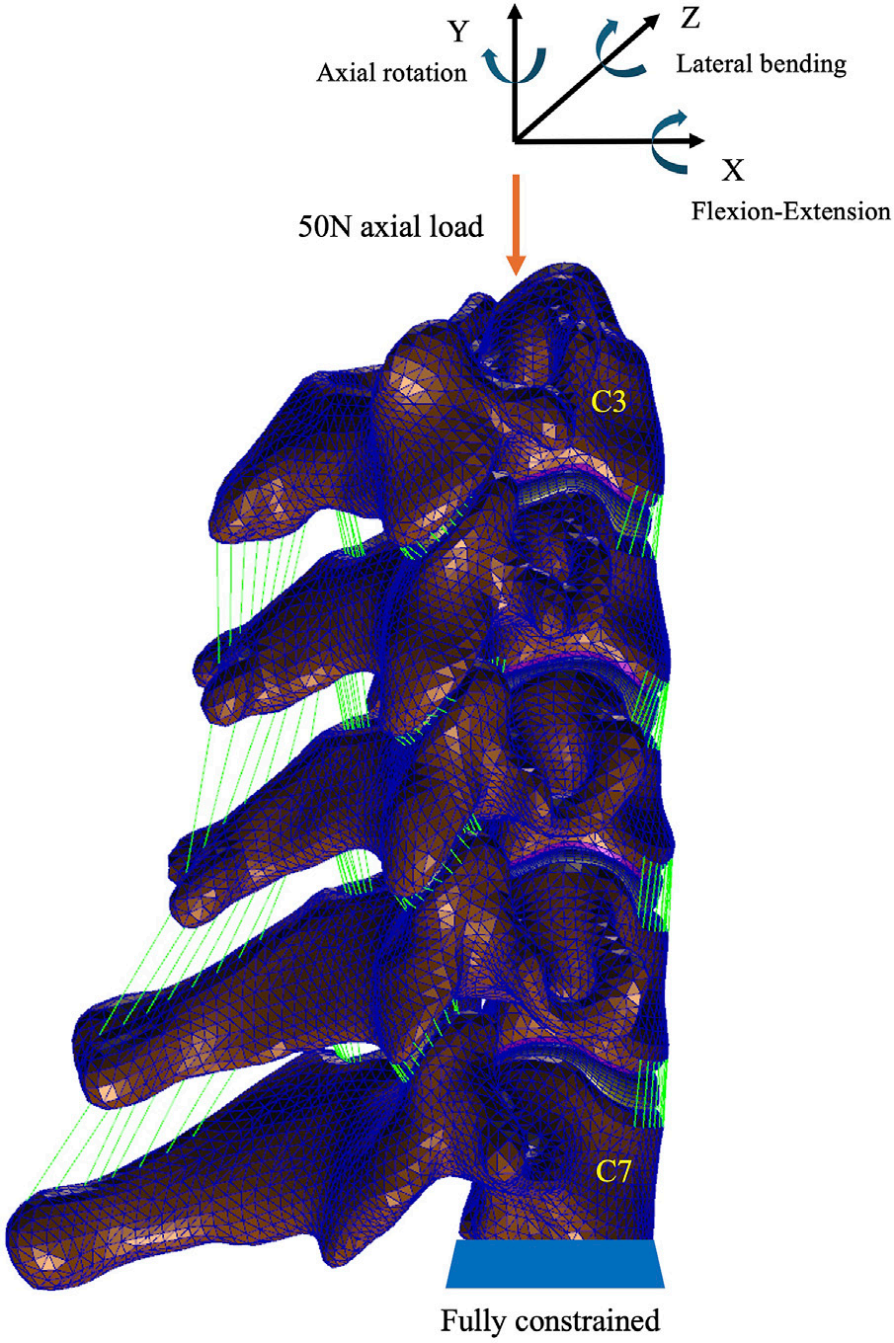
Loading and boundary conditions of the C3–C7 cervical model.

A pure axial load of 50 N with a pure moment of 1 N m was applied to the nodes connected to the upper endplate of C3 to simulate various postures (flexion-extension, lateral bending, and axial rotation) according to previous studies (Chen et al., 2020; Manickam et al., 2021). Fully constrained boundary conditions in all directions were applied to the inferior endplate of C7 during the flexion-extension, lateral bending, and axial rotation simulation (Figure 3).

### 2.6 Data analysis

To verify the ability of motion preservation, each segmental ROM and total C3-7 ROM of three surgical models were analyzed under the directions of flexion (FL), extension (EX), left bending (LB), right bending (RB), left axial rotation (LAR), and right axial rotation (RAR), compared with the intact cervical model. The intervertebral disc pressure (IDP), facet joint stress (FJS), and maximum von Mises stress in the cervical vertebrae and the instruments were quantified and analyzed via FE simulation to evaluate the stability of three surgical constructs.

## 3 Results

### 3.1 FE model validation

**FIGURE 4:**
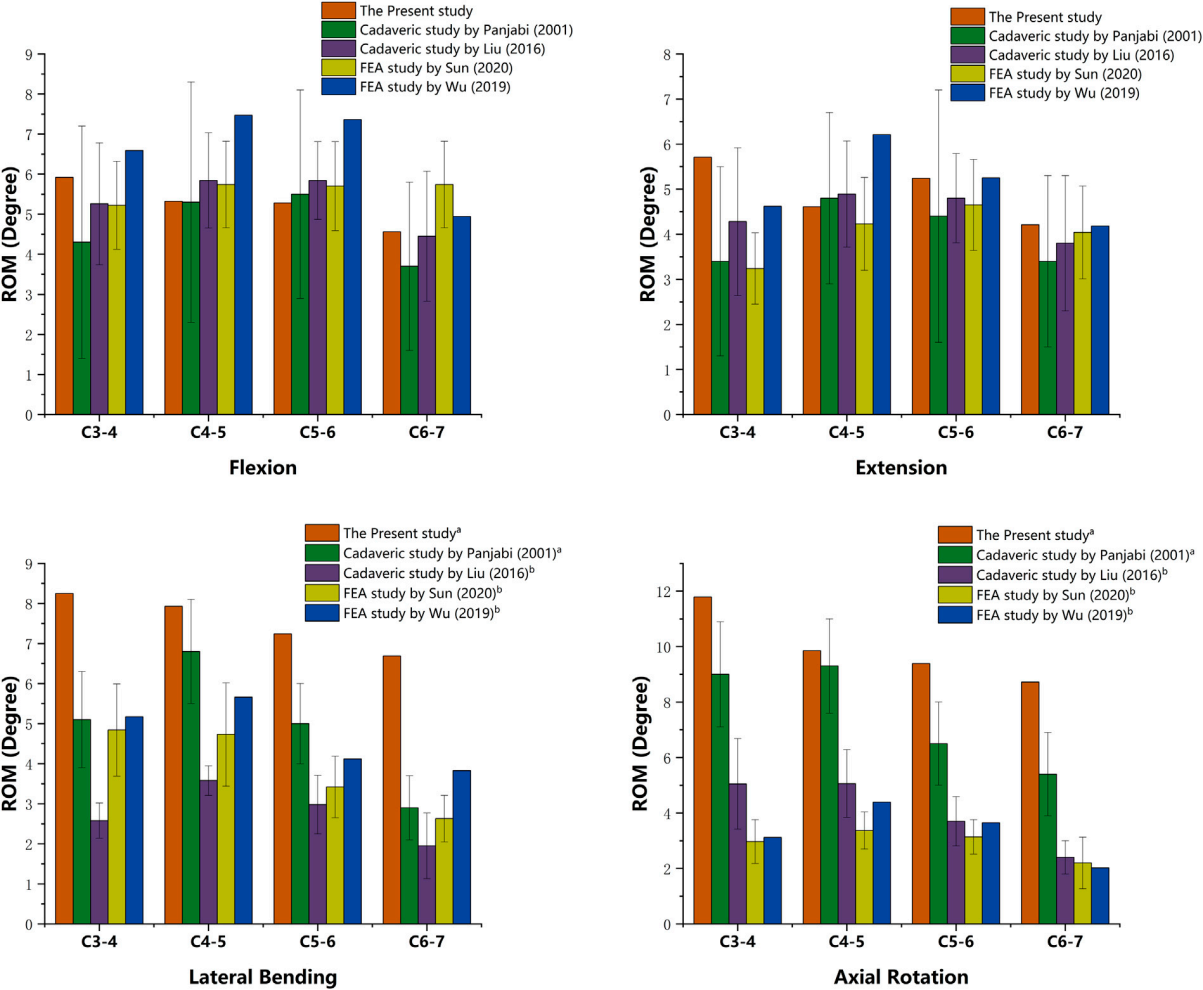
Validation of the intact cervical model. **(a)** Values of lateral bending and axial rotation summate both right and left motion. **(b)** Values of lateral bending and axial rotation are unilateral.

To affirm its validity, the intact model of the C3-C7 cervical spine was compared with prior biomechanical and FEA studies [19–22]. The segmental ROM for flexion, extension, lateral bending, and axial rotation indicates high consistency. Thus, the present subaxial cervical spine FE model was considered valid and practicable. A detailed comparison is presented in Figure 4.

### 3.2 Range of motion (ROM)

**FIGURE 5:**
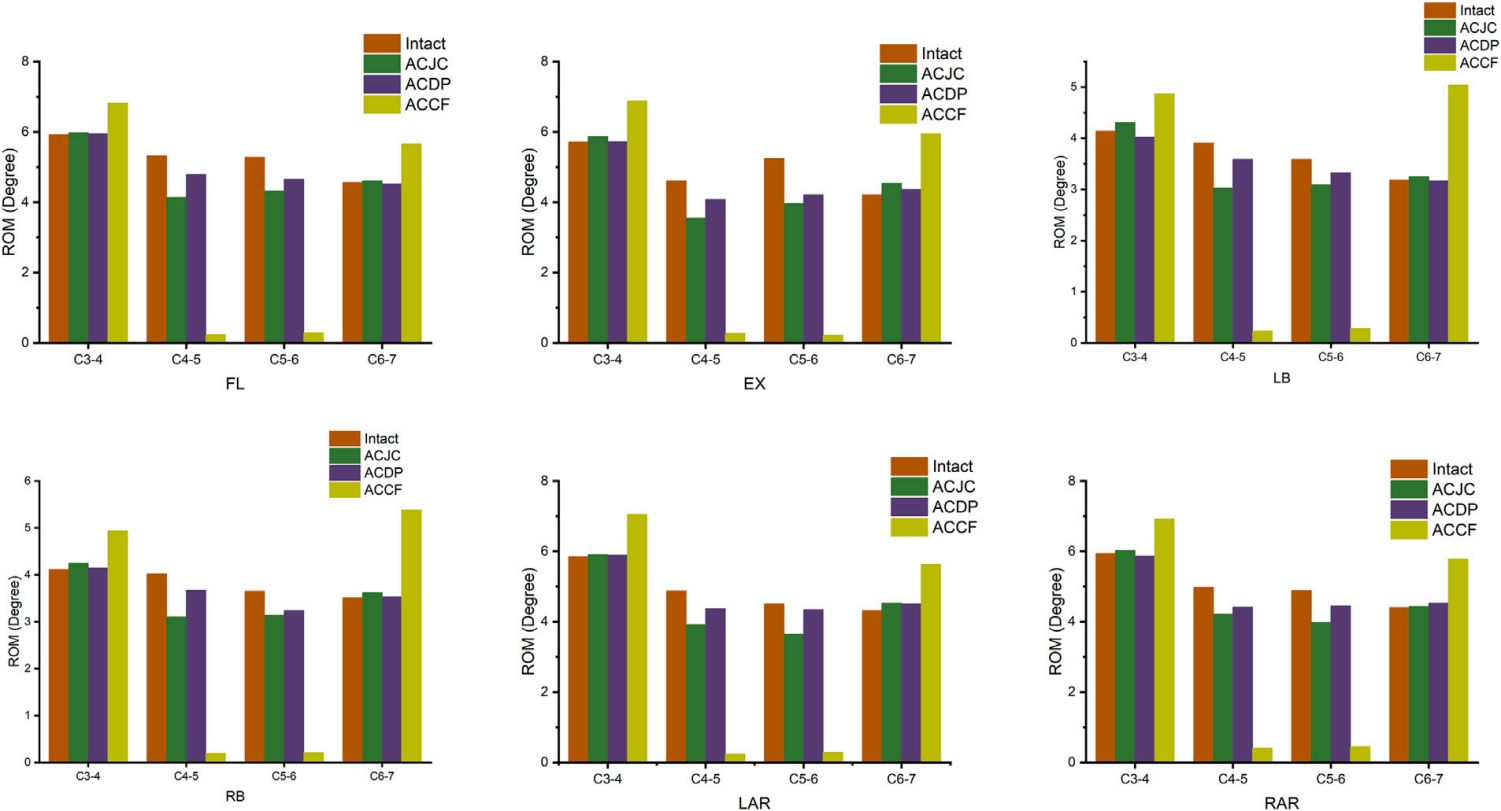
Comparison of segmental ROM between intact model and different surgical models under loading directions of FL, EX, LB, RB, LAR, and RAR (FL, flexion; EX, extension; LB, left bending; RB, right bending; LAR, left axial rotation; RAR, right axial rotation).

The comparison of the overall ROM across the three surgical constructs and the intact model under six different loading directions is illustrated in Figure 5. The ROM of the C3-C7 segments exhibited a reduction across all surgical models compared to the intact model. The ACCF model demonstrated the most significant reduction in overall mobility, showing reductions ranging from 29.7% to 38.3% compared to the intact model, with the most pronounced decreases occurring during flexion-extension and axial rotation. The CDA model maintained most of the overall motion of the subaxial cervical spine, with a tiny decline of 2.3%–7.1%, closely mirroring the intact model’s mobility. The ACJC model exhibited a small increase in ROM loss compared to the CDA model, yet it was notably less than that observed in the ACCF model. The segmental ROM of C3–C4 and C6–C7 augmented significantly across all loading directions following ACCF surgery, while such an increment was not pronounced in the ACJC and CDA interventions.

### 3.3 Intervertebral disc pressure (IDP)

**FIGURE 6:**
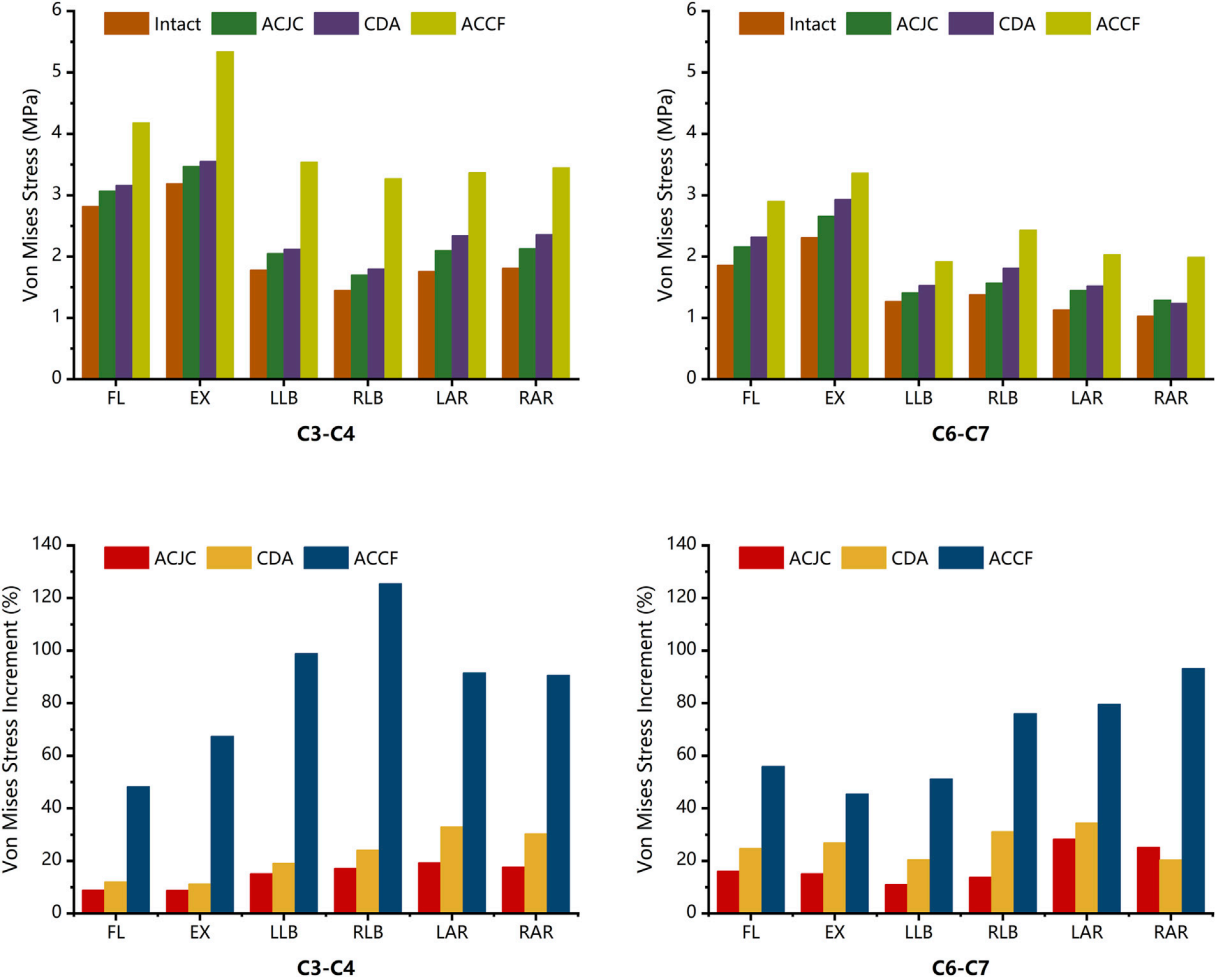
Comparison of intervertebral disc pressure and its increment between intact model and different surgical models under loading directions of FL, EX, LB, RB, LAR, and RAR (FL, flexion; EX, extension; LB, left bending; RB, right bending; LAR, left axial rotation; RAR, right axial rotation).

Figure 6 illustrates the variations in IDP at the C3-C4 and C6-C7 levels under different motion states, that all surgical constructs surpassed the intact model in IDP values at adjacent segments. The ACCF model demonstrated a notable increment in IDP at C3-C4 and C6-C7 compared to the intact model, particularly marked during lateral bending at the C3-C4 level and axial rotations at the C6-C7 level. The ACJC and CDA models imposed less impact on the C3-C4 disc, with IDP increases approximately between 8.8% and 19.3% for ACJC and 11.3%–33.0% for CDA, with the latter presenting a slightly higher increment in C3-C4 disc stress. A similar pattern of IDP changes was noted at the C6-C7 level in both ACJC and CDA models.

### 3.4 Facet joint stress (FJS)

**FIGURE 7:**
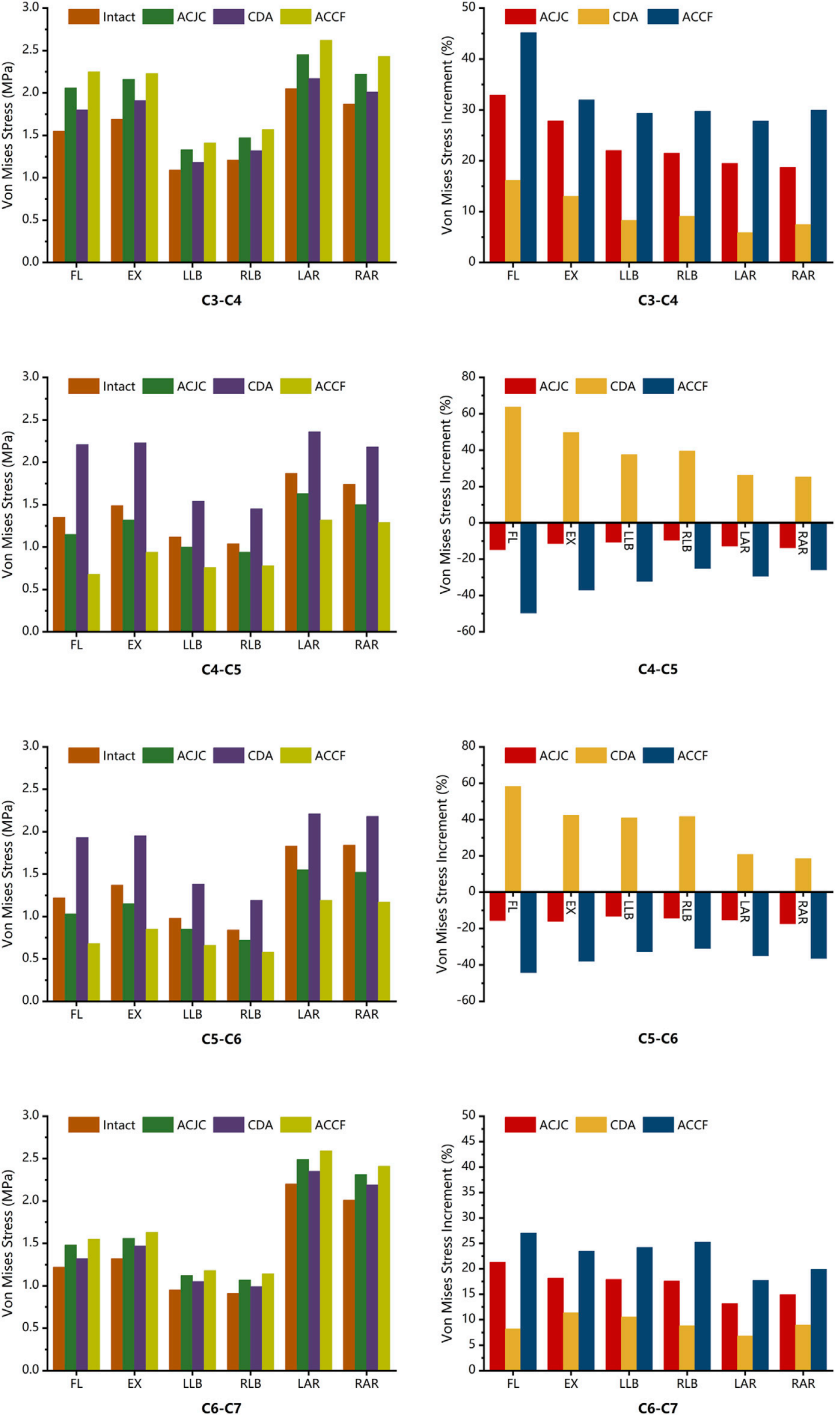
Comparison of FJS and its increment between intact model and different surgical models under loading directions of FL, EX, LB, RB, LAR, and RAR (FL, flexion; EX, extension; LB, left bending; RB, right bending; LAR, left axial rotation; RAR, right axial rotation).

Simulated analyses of each segment’s facet joint stress (FJS) were conducted separately. Overall, the index segments in the CDA model displayed the highest FJS increment, while ACCF showed the lowest. In contrast, increased FJS was observed in the adjacent segments in ACCF and ACJC models. Notably, in the CDA model, the FJS in C4-C5 and C5-C6 levels experienced significant elevations, with increases relative to the intact model ranging from 25.3% to 63.7% and 18.5%–58.2%, respectively, especially during flexion and extension. The ACJC and ACCF models exhibited FJS reductions in C4-C5 and C5-C6 levels compared to the intact model, with the most considerable decrease during flexion and extension. At the C3-C4 and C6-C7 levels, FJS rose across all surgical models to various extents. The ACCF model showed the most pronounced increase during flexion, with the FJS at C3-C4 and C6-C7 ascending by 45.2% and 27%, respectively, compared to the intact model. The ACJC model also manifested an FJS increase, particularly in flexion, with a rise of 32.9% and 21.3% in the stress of the upper and lower adjacent segments, respectively. Relatively minor FJS increments were observed at the C3-C4 and C6-C7 levels. The specifics are detailed in Figure 7.

### 3.5 Maximum von Mises stress on the cervical vertebrae and instruments

**FIGURE 8:**
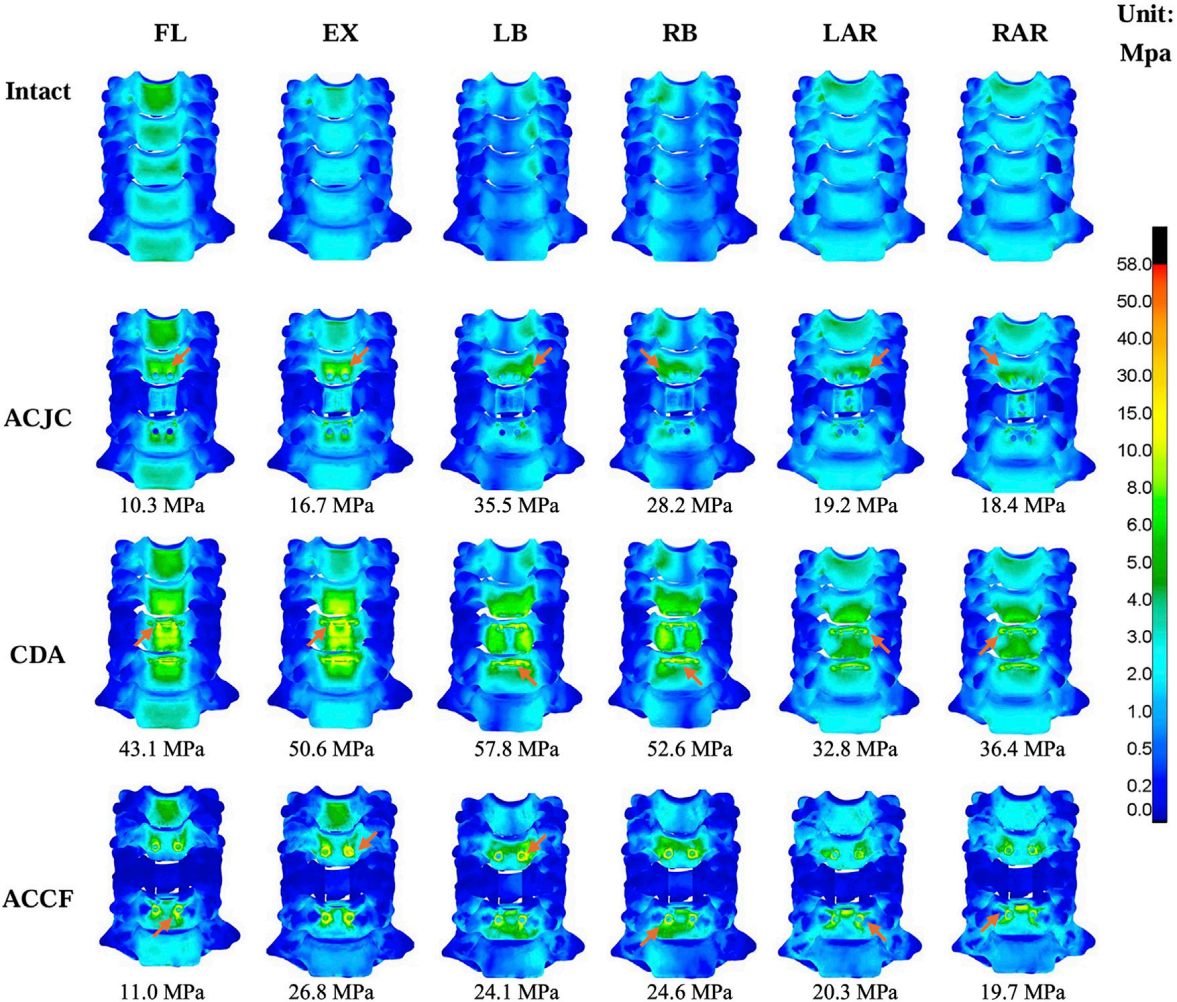
Comparison of the maximum von Mises stress and the stress distribution of vertebrae between intact and different surgical models under loading directions of FL, EX, LB, RB, LAR and RAR (FL, flexion; EX, extension; LB, left bending; RB, right bending; LAR, left axial rotation; RAR, right axial rotation), the arrow points to the area of maximum stress.

The maximum vertebral von Mises stress and its distribution in different models are illustrated in Figure 8. In the six motion states, all ACJC prosthesis, ACDP, and TCM-plate-screws system influenced adjacent vertebral stress. In the CDA model, the C5 vertebra showed the highest stress increase during flexion-extension and lateral bending, ranging from 43.1 MPa to 57.8 MPa. The ACCF model exhibited the slightest variation in vertebrae von Mises stress of 11.0 MPa–26.8 MPa, primarily concentrated around the screw-contact regions in C4 and C6 vertebrae. The ACJC prosthesis prompted a stress rise in the C4 and C6 vertebrae during lateral bending, whereas it was less marked during flexion-extension and axial rotation. The AJCJ model showed a medium von Mises stress variation on adjacent vertebrae compared to the ACCF and CDA models.

**FIGURE 9:**
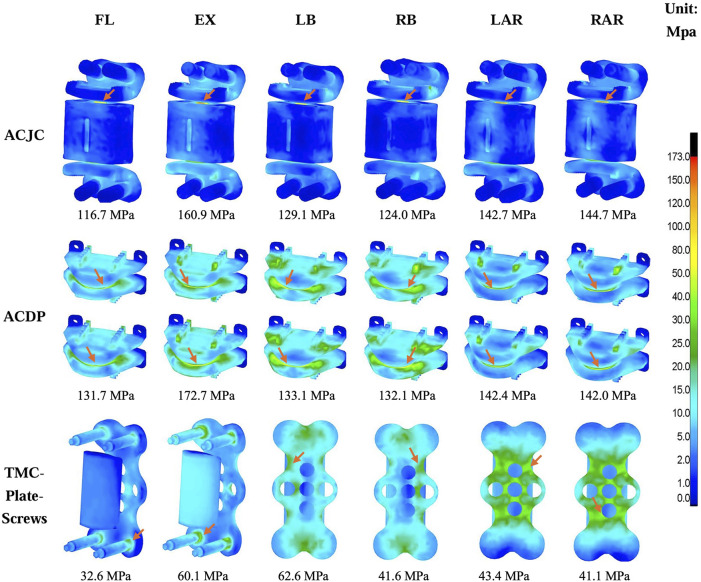
Comparison of the maximum von Mises stress and the stress distribution between different instruments models under loading directions of FL, EX, LB, RB, LAR, and RAR (FL, flexion; EX, extension; LB, left bending; RB, right bending; LAR, left axial rotation; RAR, right axial rotation), the arrow points to the area of maximum stress.

Figure 9 present the maximum von Mises stress values and their distribution in each instrument model. The ACJC prosthesis and ACDP experienced maximal stress during extension, predominantly localized at the joint interfaces, measuring 160.9 MPa and 172.7 MPa, respectively. The stress in the TCM-plate-screws system was comparatively lower across all motion states and mainly distributed at the root of the screws.

## 4 Discussion

Anterior cervical fusion surgery stands as the primary management of cervical disorders due to the directly relieving spinal cord compression, restoring intervertebral height, correcting cervical kyphotic deformity, and reconstructing spinal stability (Davies et al., 2017). However, adjacent segment degeneration (ASD) post-fusion procedure emerges as a significant clinical challenge (Cho and Riew, 2013; Rudisill et al., 2022; Toci et al., 2022). Various studies corroborate that cervical disc arthroplasty (CDA), compared to anterior cervical discectomy and fusion (ACDF), significantly reduces the incidence of postoperative ASD (Findlay et al., 2018; Kim et al., 2023; Lee et al., 2023). Yet, no motion-preservation device has been approved for clinical use in treating cervical spondylotic myelopathy (CSM) with vertebral-body-level spinal cord compression. Our group has developed various artificial cervical joint complex (ACJC) prostheses as an alternative to cervical corpectomy and fusion (ACCF) in the context of motion preservation (Wu et al., 2012; Tan et al., 2015). The prior *in vitro* biomechanical assessments indicate that ACJC possesses effective motion preservation capabilities and a small impact on adjacent segments. Dong et al. (2015), Dong et al. (2016) also engineered different types of ACJC prostheses that were distinct from our prostheses in prosthesis material selection, the design of the joint structure, and the bone-prosthesis interface yet share similar biomechanical characteristics via *in vitro* researches. Still, stability reconstruction remained a challenge for ACJC, and few biomechanical studies comprehensively analyzed the differences between ACJC, ACCF arthroplasty, and CDA regarding postoperative segment stability. The current study further refined the ACJC prosthesis guided by the principle of physiological reconstruction and investigated whether the innovative ACJC achieves a biomechanical balance between mobility and stability following cervical corpectomy by comparing it with ACCF and two-level CDA procedures through finite element analysis (FEA).

FEA is an effective computational tool for biomechanical studies that employs data simulation to evaluate and predict the biomechanical behavior of various clinical scenarios and orthopedic instruments. It is widely utilized in the research and development of innovative spinal instrumentation. In the current study, the subaxial cervical spine (C3-C7) was selected as the subject to construct the intact model according to published FEA literature (Cai et al., 2020; Srinivasan et al., 2021; Huang et al., 2023). The anterior cervical surgical models were established at C4-C6 levels not only because the central position of the C5 vertebra within the lower cervical spine facilitates the biomechanical simulation and analysis but also because the C4-C5 and C5-C6 are most frequently implicated in disc degeneration and herniation (Jiang et al., 2011). Model validation is an essential process before the FEA to confirm its effectivity and feasibility. The current standard for validation involves cross-referencing the ROM values with existing *in vitro* studies and FEA research (Ke et al., 2021; Li et al., 2021). The obtained ROM values of the current intact FE model were aligned with the data obtained from previous cadaveric studies (Panjabi et al., 2001; Liu et al., 2016) and FE models (Wu et al., 2019; Sun et al., 2020). This alignment suggests that the present FE model was well-established and ready for subsequent biomechanical analysis.

### 4.1 Range of motion (ROM)

The etiology of ASD remains controversial, yet the prevailing view attributes it to alterations in spinal kinematics following fusion (Wu et al., 2018; Huang et al., 2023). Lower postoperative ROM is generally associated with greater enhanced stability and diminished potential for implant loosening. However, it is critical to recognize that rigid fixation may induce compensatory load increases and hasten degeneration in the adjacent segments (Galivanche et al., 2020; Ji et al., 2020). Results of the present study indicated that postoperative ROM decreases following three different anterior surgeries, with the ACCF displaying the most significant ROM loss compared to the intact model, yet CDA maintaining the ROM close to the intact model, aligning with findings reported *in vitro* experiments and FEA (Nunley et al., 2018; Patwardhan and Havey, 2020; Roch et al., 2020). The greatest ROM loss was observed in ACCF of up to 38.3% flexion reduction and over 30% reductions in extension and axial rotation. This led to significant compensatory motion at the C3-C4 and C6-C7 segments during flexion-extension and rotation, as illustrated in Figure 5. The ACJC arthroplasty and CDA exhibited similar ROM variation patterns. There was no notable increasing activity at the C3-C4 and C6-C7 segments, suggesting a relatively small impact on adjacent segments of the ACJC prosthesis that fulfilled its purpose as motion-preservation devices. Although ACJC arthroplasty did not match the mobility at C4-5 and C5-6 levels in the two-level CDA model, the redesign prosthesis was engineered to augment the stability by incorporating a semi-constrained internal joint and a novel external joint structure. Hence, a comparatively lower motion-preservation capability than ACDP was expected.

### 4.2 Maximal von Mises stress on adjacent intervertebral disc

Non-physiological activities after fusion procedures commonly lead to abnormal variations in intervertebral disc pressure (IDP) at the adjacent segments, as previously reported in cadaveric and FEA studies (Chang et al., 2007a; Park et al., 2014; Welke et al., 2016; Purushothaman et al., 2020). The cadaveric investigation by Park et al. (2014) showed a significant increase of pressure at the center of the superior adjacent disc and the anterior part of the inferior adjacent disc during flexion and axial rotation after two-level cervical fusion surgery, while the IDP remained unchanged following two-level CDA procedure. Likewise, *in vitro* biomechanical research by Welke et al. (2016) demonstrated a notable increase in kinematics and pressures at adjacent segments in fusion models, whereas no such changes were observed in CDA models. Through FEA, Purushothaman et al. (2020) found that fusion surgery significantly increased adjacent IDP during flexion, extension, and axial rotation, correlating with the notable increased ROM in adjacent segments. In the current study, IDP was evaluated within the intact model and ACJC, ACCF, and two-level CDA models, resulting in analogous findings. Variations in IDP under different loading directions are illustrated in Figure 6. Compared to the intact model, the most notable increase in IDP at C3-C4 was observed in the ACCF model during LB and RB loading with increments ranging from 98.9% to 125.5%, while significant IDP increases at C6-C7 were observed during LAR and RAR loading ranging from 79.6% to 93.2%. The ACJC and CDA models exhibited significantly lower impacts on adjacent segment discs under different loading directions than the fusion model. During axial rotation loading, both motion-preservation devices demonstrated a relative increase in IDP, potentially attributable to the decreased stability of adjacent segments caused by the excessive rotational flexibility of prostheses, in which the impact on the lower adjacent segment was greater than on the upper one, aligning with results from previous *in vitro* study (Park et al., 2014).

### 4.3 Maximal von Mises stress on facet joints

Abnormal hypermobility of facet joints at adjacent segments has been recognized as a factor contributing to ASD. Studies indicate that facet joint stress (FJS) of the non-fused segments significantly increases following fusion surgery, often accompanied by abnormal increases in ROM (Chang et al., 2007a; Li et al., 2015; Liang et al., 2022). Similar findings were observed in the present study, as illustrated in Figure 7. In the CDA model, minimal variations in FJS at adjacent segments were observed compared to the intact model, consistent with the negligible compensatory ROM at the C3-C4 and C6-C7 levels. However, the ACCF model showed a marked increase in FJS in the non-fused segments under various loading directions, with the most significant increase noted during flexion. This increase may be attributed to the restriction of flexion at the fused segments by the anterior plate. The ACJC model also showed an FJS increase at C3-C4 and C6-C7 levels during flexion-extension but significantly less than that observed in the fusion model. It was noted that the impact on adjacent segments varied with the different loading directions. Specifically, during axial rotation, the ACJC prosthesis processed the least influence on adjacent segments FJS but a greater impact on the adjacent intervertebral discs while showing the opposite effect in flexion and extension, potentially due to its endplate design and unrestricted rotational mobility. Overall, attributed to its preservation of index segment mobility, ACJC exerts a significantly lower impact on adjacent segments’ stability than fusion surgery, which could effectively reduce the incidence of ASD.

However, enhanced postoperative mobility may not always be beneficial. The cervical degeneration and the removal of critical structures such as ligaments and intervertebral discs during surgery can significantly compromise the stability of index segments (Choi et al., 2021). Rigid fixation provided by ACCF is aimed to offset these adverse effects. The Prestige-LP prosthesis used in the current study is an FDA-approved artificial cervical disc featuring an open two-piece, semi-constrained design with metal-on-metal articulation (Traynelis, 2005). Previous biomechanical investigations suggested that its semi-constrained mobility (0–10° of flexion-extension and lateral bending, up to 2 mm of anterior-posterior translation) significantly increases facet joint stress at the operative levels (Chang et al., 2007b; Choi et al., 2020). It has been presumed that such mobility may alter compressive and shear stress within the index segments, coupled with other factors such as mismatched prosthesis height, improper prosthesis placement, and excessive endplate damage, may contribute to the development of the occurrence of heterotopic ossification post-CDA (Ganbat et al., 2014; Qi et al., 2014; Shen et al., 2021). This can result in a reduction of postoperative cervical ROM, which contradicts the objectives of motion-preservation devices. Therefore, CDA is contraindicated for patients with spinal instability and facet joint disorder in current clinical practice. One of the purposes of developing the ACJC prosthesis was to address postoperative segmental instability after motion preservation surgeries. Results from the present FE simulation demonstrate that the ACJC arthroplasty significantly reduced FJS at the index levels compared with CDA. Although no excessive activity in the index segments was observed in the CDA model, there was a notable FJS augmentation in the surgical levels during flexion and extension, which may be attributed to the resection of ligaments and the excessive translation of the Prestige-LP. It is noteworthy that physiological activities of the cervical spine do include moderate translational movement. Although the novel ACJC was specifically designed to fully restrict translation at individual joints, the integrated motion between the superior and inferior joints can replicate complex cervical physiological movements. The FE models revealed that this coupled motion provides greater stability than the direct translation of the Prestige-LP. Additionally, the cervical anterior column reconstruction with the vertebral component of ACJC also contributed to enhanced postoperative stability at the index levels.

### 4.4 Maximum von Mises stress on the cervical vertebrae and instruments

Given the stability concern, the present FE study also examined the stress responses within the constructs of ACJC, ACDP, and TMC-plate-screw systems. The findings revealed that across a variety of loading scenarios, the maximum von Mises stress of C3-C7 vertebrae ranged approximately from 10.3 MPa to 57.8 MPa and remained well within the yield stress of human cortical bone (188.51 MPa) (Albert et al., 2021). As for the three implants, the observed maximum von Mises stress was approximately between 32.6 MPa and 172.7 MPa, significantly below the yield stress of the Ti6Al4V alloy (870 MPa) (Bundy et al., 1983). Consequently, no instances of vertebral fractures or structural damage to the implants were observed during the loading tests. During the simulations, the maximum von Mises stress of the cervical vertebrae was observed in the CDA model during lateral bending, as shown in Figure 8. Stress concentration was primarily noted at C5 and C6, the lower adjacent segments to the ACDP implant. This stress concentration might be attributed to the rigid metal-on-metal structure of the Prestige-LP, which may contribute to the postoperative subsidence of the prosthesis (Choi et al., 2020). Moreover, the primary stresses on the ACDP implant were noted at the articulating surfaces, suggesting the potential generation of wear debris during disc movement, which may lead to subsequent aseptic inflammation and instrumental failure eventually. In the ACCF surgical model, stress concentration was predominantly noted at the screw-plate interfaces, a factor potentially contributing to postoperative complications such as implant loosening, fractures, and fusion failure. However, the mechanical stress transmission to adjacent vertebral bodies was minimal in the ACCF model, consistent with findings reported in the literature (Fogel et al., 2003; Li et al., 2021; Oda et al., 2022). The ACJC prosthesis demonstrated von Mises stress values comparable to the ACDP’s. However, acting like a TMC, the vertebral component effectively dissipated stress and exerted significantly less impact on the adjacent vertebrae than ACDP, theoretically reducing the risk of implant collapse. However, the stress within the ACJC prosthesis was primarily concentrated at the interface between the CoCrMo joint ball and the UHMWPE socket, which could be the potential failure point of ACJC. The fatigue resistance at this critical juncture warrants further research for definitive validation.

## 5 Limitation

Taken together, the present FEA results of IDP, FJS, and the stress distribution in both implants and cervical vertebrae at index and adjacent segments in the ACJC model collectively indicated maintenance of stability post ACJC arthroplasty. However, certain limitations of the current study were noticed. Firstly, while the employed FE model has been validated, it was developed using geometric data from the cervical spine of a single healthy individual, in which the vertebrae shape may impact the simulation. Secondly, this FE model did not account for the influence of the paravertebral musculature and the variations of bone material properties in different the cervical segments (Al-Barghouthi et al., 2020; Garay et al., 2022), and the intervertebral disc has been modeled as an elastic material instead of the poroelastic medium (Volz et al., 2022), introducing the potential for an oversimplification that may not accurately simulate the stress distribution. Future FE studies should refine the simulation of cervical structures to mirror clinical reality more accurately. Thirdly, CDA surgery is not indicated for patients requiring cervical subtotal corpectomy. To facilitate comparison with the ACJC and ACCF on postoperative mobility and stability, the present FE model was established based on CT data from a healthy individual, which did not account for the potential impacts of cervical degeneration on outcomes. Additionally, to establish a full fusion model and simplify the calculations, the vertebral screws were modeled in a cylindrical shape with no threads, leading to the neglect of the potential impact of threads on cancellous bone, which was inconsistent with the clinically realistic and potentially affecting the results of the simulations. Also, as with other FE studies, the current results were generated from a simplified biomechanical analysis that may not comprehensively and accurately reflect the characteristics of the cervical spine, requiring further validation through cadaveric studies.

## 6 Conclusion

Biomechanical findings of FEA indicated that, theoretically, the ACJC addressed the issue of the rigidity associated with ACCF and the instability with CDA and effectively balanced postoperative stability with cervical motion preservation. This novel joint-structured cervical prosthesis presents a feasible alternative to ACCF as a physiological spinal reconstruction device.

## Data Availability

The original contributions presented in the study are included in the article/Supplementary Material, further inquiries can be directed to the corresponding author.

## References

[B1] Al-Barghouthi A. Lee S. Solitro G. F. Latta L. Travascio F. (2020). Relationships among bone morphological parameters and mechanical properties of cadaveric human vertebral cancellous bone. JBMR Plus 4, e10351. 10.1002/jbm4.10351 37780057 PMC10540741

[B2] Albert D. L. Katzenberger M. J. Agnew A. M. Kemper A. R. (2021). A comparison of rib cortical bone compressive and tensile material properties: trends with age, sex, and loading rate. J. Mech. Behav. Biomed. Mater 122, 104668. 10.1016/j.jmbbm.2021.104668 34265671

[B3] Bandyopadhyay A. Mitra I. Avila J. D. Upadhyayula M. Bose S. (2023). Porous metal implants: processing, properties, and challenges. Int. J. Extrem. Manuf. 5, 032014. 10.1088/2631-7990/acdd35 37476350 PMC10355163

[B4] Bhattacharya S. Goel V. K. Liu X. Kiapour A. Serhan H. A. (2011). Models that incorporate spinal structures predict better wear performance of cervical artificial discs. Spine J. 11, 766–776. 10.1016/j.spinee.2011.06.008 21802999

[B5] Bundy K. J. Marek M. Hochman R. F. (1983). *In vivo* and *in vitro* studies of the stress-corrosion cracking behavior of surgical implant alloys. J. Biomed. Mater Res. 17, 467–487. 10.1002/jbm.820170307 6863350

[B6] Cai X.-Y. Sang D. Yuchi C.-X. Cui W. Zhang C. Du C.-F. (2020). Using finite element analysis to determine effects of the motion loading method on facet joint forces after cervical disc degeneration. Comput. Biol. Med. 116, 103519. 10.1016/j.compbiomed.2019.103519 31710870

[B7] Chang U.-K. Kim D. H. Lee M. C. Willenberg R. Kim S.-H. Lim J. (2007a). Changes in adjacent-level disc pressure and facet joint force after cervical arthroplasty compared with cervical discectomy and fusion. J. Neurosurg. Spine 7, 33–39. 10.3171/SPI-07/07/033 17633485

[B8] Chang U.-K. Kim D. H. Lee M. C. Willenberg R. Kim S.-H. Lim J. (2007b). Range of motion change after cervical arthroplasty with ProDisc-C and prestige artificial discs compared with anterior cervical discectomy and fusion. J. Neurosurg. Spine 7, 40–46. 10.3171/SPI-07/07/040 17633486

[B9] Chen C. Yuchi C. X. Gao Z. Ma X. Zhao D. Li J. W. (2020). Comparative analysis of the biomechanics of the adjacent segments after minimally invasive cervical surgeries versus anterior cervical discectomy and fusion: a finite element study. J. Orthop. Transl. 23, 107–112. 10.1016/j.jot.2020.03.006 PMC7322474 32642425

[B10] Chen Z. Liu B. Dong J. Feng F. Chen R. Xie P. (2016). Comparison of anterior corpectomy and fusion versus laminoplasty for the treatment of cervical ossification of posterior longitudinal ligament: a meta-analysis. FOC 40, E8. 10.3171/2016.3.FOCUS15596 27246491

[B11] Cho S. K. Riew K. D. (2013). Adjacent segment disease following cervical spine surgery. J. Am. Acad. Orthop. Surg. 21, 3–11. 10.5435/JAAOS-21-01-3 23281466

[B12] Choi H. Purushothaman Y. Baisden J. Yoganandan N. (2020). Unique biomechanical signatures of Bryan, Prodisc C, and Prestige LP cervical disc replacements: a finite element modelling study. Eur. Spine J. 29, 2631–2639. 10.1007/s00586-019-06113-y 31606816

[B13] Choi H. Purushothaman Y. Baisden J. L. Rajasekaran D. Jebaseelan D. Yoganandan N. (2021). Comparative finite element modeling study of anterior cervical arthrodesis versus cervical arthroplasty with bryan disc or prodisc C. Mil. Med. 186, 737–744. 10.1093/milmed/usaa378 33499493

[B14] Dai H. Liu Y. Han Q. Zhang A. Chen H. Qu Y. (2022). Biomechanical comparison between unilateral and bilateral percutaneous vertebroplasty for osteoporotic vertebral compression fractures: a finite element analysis. Front. Bioeng. Biotechnol. 10, 978917. 10.3389/fbioe.2022.978917 36159704 PMC9495612

[B15] Davies B. M. McHugh M. Elgheriani A. Kolias A. G. Tetreault L. Hutchinson P. J. A. (2017). The reporting of study and population characteristics in degenerative cervical myelopathy: a systematic review. PLoS One 12, e0172564. 10.1371/journal.pone.0172564 28249017 PMC5332071

[B16] Dong J. Lu M. Liang B. Zhai X. Qin J. He X. (2016). Anterior cervical corpectomy non-fusion model produced by a novel implant. Med. Sci. Monit. 22, 1131–1145. 10.12659/msm.897244 27049839 PMC4825879

[B17] Dong J. Lu M. Lu T. Liang B. Xu J. Qin J. (2015). Artificial disc and vertebra system: a novel motion preservation device for cervical spinal disease after vertebral corpectomy. Clin. (Sao Paulo) 70, 493–499. 10.6061/clinics/2015(07)06 PMC4496753 26222819

[B18] Findlay C. Ayis S. Demetriades A. K. (2018). Total disc replacement versus anterior cervical discectomy and fusion: a systematic review with meta-analysis of data from a total of 3160 patients across 14 randomized controlled trials with both short- and medium-to long-term outcomes. Bone Jt. J. 100-B, 991–1001. 10.1302/0301-620X.100B8.BJJ-2018-0120.R1 30062947

[B19] Fogel G. R. Reitman C. A. Liu W. Esses S. I. (2003). Physical characteristics of polyaxial-headed pedicle screws and biomechanical comparison of load with their failure. Spine (Phila Pa 1976) 28, 470–473. 10.1097/01.BRS.0000048652.45964.2E 12616159

[B20] Galivanche A. R. Gala R. Bagi P. S. Boylan A. J. Dussik C. M. Coutinho P. D. (2020). Perioperative outcomes in 17,947 patients undergoing 2-level anterior cervical discectomy and fusion versus 1-level anterior cervical corpectomy for treatment of cervical degenerative conditions: a propensity score matched national surgical quality improvement program analysis. Neurospine 17, 871–878. 10.14245/ns.2040134.067 33401865 PMC7788425

[B21] Ganbat D. Kim K. Jin Y. J. Kim Y. H. (2014). Heterotopic ossification in cervical total disk replacement: a finite element analysis. Proc. Inst. Mech. Eng. H. 228, 200–205. 10.1177/0954411914522024 24477889

[B22] Garay R. S. Solitro G. F. Lam K. C. Morris R. P. Albarghouthi A. Lindsey R. W. (2022). Characterization of regional variation of bone mineral density in the geriatric human cervical spine by quantitative computed tomography. PLoS ONE 17, e0271187. 10.1371/journal.pone.0271187 35802639 PMC9269429

[B23] Guo X. Zhou J. Tian Y. Kang L. Xue Y. (2021). Biomechanical effect of different plate-to-disc distance on surgical and adjacent segment in anterior cervical discectomy and fusion - a finite element analysis. BMC Musculoskelet. Disord. 22, 340. 10.1186/s12891-021-04218-4 33836709 PMC8035773

[B24] Hua W. Zhi J. Ke W. Wang B. Yang S. Li L. (2020a). Adjacent segment biomechanical changes after one- or two-level anterior cervical discectomy and fusion using either a zero-profile device or cage plus plate: a finite element analysis. Comput. Biol. Med. 120, 103760. 10.1016/j.compbiomed.2020.103760 32421657

[B25] Hua W. Zhi J. Wang B. Ke W. Sun W. Yang S. (2020b). Biomechanical evaluation of adjacent segment degeneration after one- or two-level anterior cervical discectomy and fusion versus cervical disc arthroplasty: a finite element analysis. Comput. Meth Prog. Bio 189, 105352. 10.1016/j.cmpb.2020.105352 31991316

[B26] Huang G. Pan S.-T. Qiu J.-X. (2022). The osteogenic effects of porous Tantalum and Titanium alloy scaffolds with different unit cell structure. Colloids Surf. B Biointerfaces 210, 112229. 10.1016/j.colsurfb.2021.112229 34875470

[B27] Huang S. Ling Q. Lin X. Qin H. Luo X. Huang W. (2023). Biomechanical evaluation of a novel anterior transpedicular screw-plate system for anterior cervical corpectomy and fusion (ACCF): a finite element analysis. Front. Bioeng. Biotechnol. 11, 1260204. 10.3389/fbioe.2023.1260204 38026869 PMC10665523

[B28] Hui N. Phan K. Kerferd J. Lee M. Mobbs R. J. (2020). Prevalence of and risk factors for heterotopic ossification after cervical total disc replacement: a systematic review and meta-analysis. Glob. Spine J. 10, 790–804. 10.1177/2192568219881163 PMC7383784 32707022

[B29] Ji C. Yu S. Yan N. Wang J. Hou F. Hou T. (2020). Risk factors for subsidence of titanium mesh cage following single-level anterior cervical corpectomy and fusion. BMC Musculoskelet. Disord. 21, 32. 10.1186/s12891-019-3036-8 31937288 PMC6961320

[B30] Jiang S.-D. Jiang L.-S. Dai L.-Y. (2011). Degenerative cervical spondylolisthesis: a systematic review. Int. Orthop. 35, 869–875. 10.1007/s00264-010-1203-5 21264670 PMC3103955

[B31] Ke W. Chen C. Wang B. Hua W. Lu S. Song Y. (2021). Biomechanical evaluation of different surgical approaches for the treatment of adjacent segment diseases after primary anterior cervical discectomy and fusion: a finite element analysis. Front. Bioeng. Biotechnol. 9, 718996. 10.3389/fbioe.2021.718996 34532313 PMC8438200

[B32] Kim L. J. Y. Mazur M. D. Dailey A. T. (2023). Mid-term and long-term outcomes after total cervical disk arthroplasty compared with anterior cervical discectomy and fusion: a systematic review and meta-analysis of randomized controlled trials. Clin. Spine Surg. 36, 339–355. 10.1097/BSD.0000000000001537 37735768

[B33] Latka D. Kozlowska K. Miekisiak G. Latka K. Chowaniec J. Olbrycht T. (2019). Safety and efficacy of cervical disc arthroplasty in preventing the adjacent segment disease: a meta-analysis of mid-to long-term outcomes in prospective, randomized, controlled multicenter studies. Ther. Clin. Risk Manag. 15, 531–539. 10.2147/TCRM.S196349 30992666 PMC6445235

[B34] Lee J. H. Lee Y. J. Chang M. C. Lee J. H. (2023). Clinical effectiveness of artificial disc replacement in comparison with anterior cervical discectomy and fusion in the patients with cervical myelopathy: systematic review and meta-analysis. Neurospine 20, 1047–1060. 10.14245/ns.2346498.249 37798997 PMC10562247

[B35] Lee S.-H. Im Y.-J. Kim K.-T. Kim Y.-H. Park W.-M. Kim K. (2011). Comparison of cervical spine biomechanics after fixed- and mobile-core artificial disc replacement: a finite element analysis. Spine 36, 700–708. 10.1097/BRS.0b013e3181f5cb87 21245792

[B36] Li H. Pei B.-Q. Yang J.-C. Hai Y. Li D.-Y. Wu S.-Q. (2015). Load rate of facet joints at the adjacent segment increased after fusion. Chin. Med. J. Engl. 128, 1042–1046. 10.4103/0366-6999.155080 25881597 PMC4832943

[B37] Li Z. Liu H. Yang M. Zhang W. (2021). A biomechanical analysis of four anterior cervical techniques to treating multilevel cervical spondylotic myelopathy: a finite element study. BMC Musculoskelet. Disord. 22, 278. 10.1186/s12891-021-04150-7 33722229 PMC7962321

[B38] Liang W. Han B. Hai Y. Yang J. Yin P. (2022). Biomechanical analysis of the reasonable cervical range of motion to prevent non-fusion segmental degeneration after single-level ACDF. Front. Bioeng. Biotechnol. 10, 918032. 10.3389/fbioe.2022.918032 35782514 PMC9243332

[B39] Liu Q. Guo Q. Yang J. Zhang P. Xu T. Cheng X. (2016). Subaxial cervical intradiscal pressure and segmental kinematics following atlantoaxial fixation in different angles. World Neurosurg. 87, 521–528. 10.1016/j.wneu.2015.09.025 26409072

[B40] Manickam P. S. Roy S. Shetty G. M. (2021). Biomechanical evaluation of a novel S-type, dynamic zero-profile cage design for anterior cervical discectomy and fusion with variations in bone graft shape: a finite element analysis. World Neurosurg. 154, e199–e214. 10.1016/j.wneu.2021.07.013 34246827

[B41] McCormick J. R. Sama A. J. Schiller N. C. Butler A. J. Donnally C. J. (2020). Cervical spondylotic myelopathy: a guide to diagnosis and management. J. Am. Board Fam. Med. 33, 303–313. 10.3122/jabfm.2020.02.190195 32179614

[B42] Nunley P. D. Coric D. Frank K. A. Stone M. B. (2018). Cervical disc arthroplasty: current evidence and real-world application. Neurosurgery 83, 1087–1106. 10.1093/neuros/nyx579 29325074

[B43] Oda Y. Takigawa T. Ito Y. Misawa H. Tetsunaga T. Uotani K. (2022). Mechanical study of various pedicle screw systems including percutaneous pedicle screw in Trauma treatment. Med. Kaunas. 58, 565. 10.3390/medicina58050565 PMC9143153 35629982

[B44] Panjabi M. M. Crisco J. J. Vasavada A. Oda T. Cholewicki J. Nibu K. (2001). Mechanical properties of the human cervical spine as shown by three-dimensional load–displacement curves. Spine 26, 2692–2700. 10.1097/00007632-200112150-00012 11740357

[B45] Park J. Shin J. J. Lim J. (2014). Biomechanical analysis of disc pressure and facet contact force after simulated two-level cervical surgeries (fusion and arthroplasty) and hybrid surgery. World Neurosurg. 82, 1388–1393. 10.1016/j.wneu.2014.06.013 24937596

[B46] Patwardhan A. G. Havey R. M. (2020). Prosthesis design influences segmental contribution to total cervical motion after cervical disc arthroplasty. Eur. Spine J. 29, 2713–2721. 10.1007/s00586-019-06064-4 31309331

[B47] Pescatori L. Tropeano M. P. Visocchi M. Grasso G. Ciappetta P. (2020). Cervical spondylotic myelopathy: when and why the cervical corpectomy? World Neurosurg. 140, 548–555. 10.1016/j.wneu.2020.03.100 32797986

[B48] Purushothaman Y. Yoganandan N. Jebaseelan D. Choi H. Baisden J. (2020). External and internal responses of cervical disc arthroplasty and anterior cervical discectomy and fusion: a finite element modeling study. J. Mech. Behav. Biomed. Mater 106, 103735. 10.1016/j.jmbbm.2020.103735 32321632

[B49] Qi M. Chen H. Cao P. Tian Y. Yuan W. (2014). Incidence and risk factors analysis of heterotopic ossification after cervical disc replacement. Chin. Med. J. Engl. 127, 3871–3875. 10.3760/cma.j.issn.0366-6999.20141913 25421183

[B50] Roch P. J. Wagner M. Weiland J. Spiering S. Lehmann W. Saul D. (2020). Total disc arthroplasties alter the characteristics of the instantaneous helical axis of the cervical functional spinal units C3/C4 and C5/C6 during flexion and extension in *in vitro* conditions. J. Biomech. 100, 109608. 10.1016/j.jbiomech.2020.109608 31926589

[B51] Rudisill S. S. Hornung A. L. Barajas J. N. Bridge J. J. Mallow G. M. Lopez W. (2022). Artificial intelligence in predicting early-onset adjacent segment degeneration following anterior cervical discectomy and fusion. Eur. Spine J. 31, 2104–2114. 10.1007/s00586-022-07238-3 35543762

[B52] Shen Y.-W. Yang Y. Liu H. Rong X. Ding C. Meng Y. (2021). Effects of endplate coverage and intervertebral height change on heterotopic ossification following cervical disc replacement. J. Orthop. Surg. Res. 16, 693. 10.1186/s13018-021-02840-5 34823557 PMC8614029

[B53] Srinivasan S. Kumar S. D. Shruthi R. Jebaseelan D. D. Yoganandan N. Rajasekaran S. (2021). Effect of heterotopic ossification after bryan-cervical disc arthroplasty on adjacent level range of motion: a finite element study. J. Clin. Orthop. Trauma 15, 99–103. 10.1016/j.jcot.2020.10.027 33717922 PMC7920132

[B54] Sun J. Wang Q. Cai D. Gu W. Ma Y. Sun Y. (2021). A lattice topology optimization of cervical interbody fusion cage and finite element comparison with ZK60 and Ti-6Al-4V cages. BMC Musculoskelet. Disord. 22, 390. 10.1186/s12891-021-04244-2 33902500 PMC8077704

[B55] Sun X. Sun S. Zhang T. Kong C. Wang W. Lu S. (2020). Biomechanical comparison of noncontiguous cervical disc arthroplasty and noncontiguous cervical discectomy and fusion in the treatment of noncontinuous cervical degenerative disc disease: a finite element analysis. J. Orthop. Surg. Res. 15, 36. 10.1186/s13018-020-1549-3 32005193 PMC6995191

[B56] Tan Q. Feng Y. Zhang Y. Wu Z. Ma Z. Sang H. (2015). A novel total cervical prosthesis for single-level cervical subtotal corpectomy: radiologic and histomorphometric analysis in a caprine model. J. Spinal Disord. Tech. 28, E166–E172. 10.1097/BSD.0000000000000202 25353202

[B57] Toci G. R. Canseco J. A. Patel P. D. Divi S. N. Goz V. Shenoy K. (2022). The incidence of adjacent segment pathology after cervical disc arthroplasty compared with anterior cervical discectomy and fusion: a systematic review and meta-analysis of randomized clinical trials. World Neurosurg. 160, e537–e548. 10.1016/j.wneu.2022.01.072 35085804

[B58] Traynelis V. C. (2005). The Prestige cervical disc. Neurosurg. Clin. N. Am. 16, 621–628. 10.1016/j.nec.2005.06.001 16326285

[B59] Volz M. Elmasry S. Jackson A. R. Travascio F. (2022). Computational modeling intervertebral disc pathophysiology: a review. Front. Physiol. 12, 750668. 10.3389/fphys.2021.750668 35095548 PMC8793742

[B60] Wang H. Wang X. Liu H. Meng Y. Guo Y. Hong Y. (2021). Risk factors for high-grade heterotopic ossification after total disc replacement: a single-center experience of 394 cases. Neurosurgery 89, 852–861. 10.1093/neuros/nyab298 34382657

[B61] Welke B. Schwarze M. Hurschler C. Book T. Magdu S. Daentzer D. (2016). *In vitro* investigation of a new dynamic cervical implant: comparison to spinal fusion and total disc replacement. Eur. Spine J. 25, 2247–2254. 10.1007/s00586-015-4361-8 26684468

[B62] Wu H.-H. Tang T. Yu X. Pang Q.-J. (2018). Stability of two anterior fixations for three-column injury in the lower cervical spine: biomechanical evaluation of anterior pedicle screw-plate fixation. J. Int. Med. Res. 46, 1455–1460. 10.1177/0300060517734687 29333900 PMC6091821

[B63] Wu T. Meng Y. Wang B. Rong X. Hong Y. Ding C. (2019). Biomechanics following skip-level cervical disc arthroplasty versus skip-level cervical discectomy and fusion: a finite element-based study. BMC Musculoskelet. Disord. 20, 49. 10.1186/s12891-019-2425-3 30704444 PMC6357490

[B64] Wu Z. Han B. Zhao X. Kong L. Liu D. Cui G. (2012). Biomechanical evaluation of a novel total cervical prosthesis in a single-level cervical subtotal corpectomy model: an *in vitro* human cadaveric study. J. Surg. Res. 175, 76–81. 10.1016/j.jss.2011.02.022 21492873

[B65] Yuchi C.-X. Sun G. Chen C. Liu G. Zhao D. Yang H. (2019). Comparison of the biomechanical changes after percutaneous full-endoscopic anterior cervical discectomy versus posterior cervical foraminotomy at C5-C6: a finite element-based study. World Neurosurg. 128, e905–e911. 10.1016/j.wneu.2019.05.025 31096026

