# Supplementary material for: Does the novel artificial cervical joint complex resolve the conflict between stability and mobility after anterior cervical surgery? a finite element study
Source: Front Bioeng Biotechnol. 2024 Jun 3;12:1400614. doi: 10.3389/fbioe.2024.1400614 (PMC11180832; doi:10.3389/fbioe.2024.1400614)
Supplement: Supplementary file 1 [file Table1.docx]

# Supplementary Tables

**Table 1**

|  | **The Present study** | | | | **Cadaveric study by Panjabi(2001)** | | | | **Cadaveric study by Liu (2016)** | | | | | **FEA study by Sun (2020)** | | | | | **FEA study by Wu (2019)** | | | | |
| --- | --- | --- | --- | --- | --- | --- | --- | --- | --- | --- | --- | --- | --- | --- | --- | --- | --- | --- | --- | --- | --- | --- | --- |
|  | **FL** | **EX** | **LB^a^** | **AR^a^** | **FL** | **EX** | **LB^a^** | **AR^a^** | **FL** | **EX** | **LB^b^** | **AR^b^** | **FL** | | **EX** | **LB^b^** | **AR^b^** | **FL** | | **EX** | **LB^b^** | **AR^b^** |  |
| **C3-4** | 5.92 | 5.71 | 8.25 | 11.79 | 4.3±2.9 | 3.4±2.1 | 5.1±1.2 | 9.0±1.9 | 5.26±1.52 | 4.28±1.64 | 2.58±0.44 | 5.05±1.63 | 5.22±1.10 | | 3.24±0.79 | 4.84±1.15 | 2.97±0.79 | 6.59 | | 4.62 | 5.17 | 3.12 |  |
| **C4-5** | 5.32 | 4.61 | 7.93 | 9.85 | 5.3±3.0 | 4.8±1.9 | 6.8±1.3 | 9.3±1.7 | 5.84±1.19 | 4.89±1.18 | 3.58±0.37 | 5.06±1.22 | 5.74±1.08 | | 4.23±1.03 | 4.73±1.29 | 3.73±0.67 | 7.47 | | 6.21 | 5.66 | 4.39 |  |
| **C5-6** | 5.28 | 5.24 | 7.24 | 9.39 | 5.5±2.6 | 4.4±2.8 | 5.0±1.0 | 6.5±1.5 | 5.84±0.97 | 4.80±0.99 | 2.98±0.73 | 3.7±0.89 | 5.70±1.11 | | 4.65±1.01 | 3.42±0.77 | 3.14±0.62 | 7.36 | | 5.25 | 4.12 | 3.65 |  |
| **C6-7** | 4.56 | 4.21 | 6.69 | 8.72 | 3.7±2.1 | 3.4±1.9 | 2.9±0.8 | 5.4±1.5 | 4.45±1.62 | 3.8±1.50 | 1.95±0.82 | 2.40±0.60 | 5.74±1.08 | | 4.04±1.03 | 2.63±0.58 | 2.20±0.93 | 4.94 | | 4.18 | 3.83 | 2.02 |  |

The segmental ROM (°) under different loading directions compared with previous studies.

a. Values of lateral bending (LB) and axial rotation (AR) summate both right and left motion.

b. Values of lateral bending (LB) and axial rotation (AR) are unilateral.

**Table 2**

|  | Intact Model | | | | | | ACJC | | | | | | CDA | | | | | | ACCF | | | | | |
| --- | --- | --- | --- | --- | --- | --- | --- | --- | --- | --- | --- | --- | --- | --- | --- | --- | --- | --- | --- | --- | --- | --- | --- | --- |
|  | **FL** | **EX** | **LB** | **RB** | **LAR** | **RAR** | **FL** | **EX** | **LB** | **RB** | **LAR** | **RAR** | **FL** | **EX** | **LB** | **RB** | **LAR** | **RAR** | **FL** | **EX** | **LB** | **RB** | **LAR** | **RAR** |
| C3-4 | 5.92 | 5.71 | 4.14 | 4.11 | 5.85 | 5.94 | 5.97 | 5.87 | 4.31 | 4.25 | 5.91 | 6.02 | 5.95 | 5.72 | 4.02 | 4.15 | 5.89 | 5.87 | 6.82 | 6.88 | 4.87 | 4.94 | 7.05 | 6.92 |
| C4-5 | 5.32 | 4.61 | 3.91 | 4.02 | 4.87 | 4.98 | 4.14 | 3.55 | 3.03 | 3.1 | 3.92 | 4.21 | 4.79 | 4.08 | 3.59 | 3.67 | 4.37 | 4.41 | 0.24 | 0.27 | 0.23 | 0.19 | 0.24 | 0.41 |
| C5-6 | 5.28 | 5.24 | 3.59 | 3.65 | 4.51 | 4.88 | 4.32 | 3.96 | 3.09 | 3.14 | 3.65 | 3.98 | 4.65 | 4.21 | 3.32 | 3.24 | 4.34 | 4.45 | 0.29 | 0.22 | 0.28 | 0.21 | 0.29 | 0.45 |
| C6-7 | 4.56 | 4.21 | 3.18 | 3.51 | 4.32 | 4.4 | 4.61 | 4.54 | 3.25 | 3.62 | 4.53 | 4.43 | 4.52 | 4.36 | 3.17 | 3.53 | 4.51 | 4.53 | 5.66 | 5.95 | 5.04 | 5.38 | 5.63 | 5.78 |
| C3-7 | 21.08 | 19.77 | 14.82 | 15.29 | 19.55 | 20.2 | 19.04 | 17.92 | 13.68 | 14.11 | 18.01 | 18.64 | 19.91 | 18.37 | 14.1 | 14.59 | 19.11 | 19.26 | 13.01 | 13.32 | 10.42 | 10.72 | 13.21 | 13.56 |

Comparison of the segmental and overall ROM (°) under different loading directions in ACJC, CDA, ACCF, and intact model.

**Table 3**

Comparison of intervertebral disc pressure (MPa) in adjacent segments in ACJC, CDA, ACCF, and intact model.

|  |  | Intact | ACJC | | CDA | | | ACCF | |
| --- | --- | --- | --- | --- | --- | --- | --- | --- | --- |
|  |  | Von Mises stress (Mpa) | Von Mises stress (Mpa) | Increment  (%) | Von Mises stress (Mpa) | Increment  (%) | | Von Mises stress (Mpa) | Increment  (%) |
| C3-C4 | **FL** | 2.82 | 3.07 | 8.9 | 3.16 | | 12.1 | 4.18 | 48.2 |
|  | **EX** | 3.19 | 3.47 | 8.8 | 3.55 | | 11.3 | 5.34 | 67.4 |
|  | **LB** | 1.78 | 2.05 | 15.2 | 2.12 | | 19.1 | 3.54 | 98.9 |
|  | **RB** | 1.45 | 1.70 | 17.2 | 1.80 | | 24.1 | 3.27 | 125.5 |
|  | **LAR** | 1.76 | 2.10 | 19.3 | 2.34 | | 33.0 | 3.37 | 91.5 |
|  | **RAR** | 1.81 | 2.13 | 17.7 | 2.36 | | 30.4 | 3.45 | 90.6 |
| C6-C7 | **FL** | 1.86 | 2.16 | 16.1 | 2.32 | | 24.7 | 2.90 | 55.9 |
|  | **EX** | 2.31 | 2.66 | 15.2 | 2.93 | | 26.8 | 3.36 | 45.5 |
|  | **LB** | 1.27 | 1.41 | 11.0 | 1.53 | | 20.5 | 1.92 | 51.2 |
|  | **RB** | 1.38 | 1.57 | 13.8 | 1.81 | | 31.2 | 2.43 | 76.1 |
|  | **LAR** | 1.13 | 1.45 | 28.3 | 1.52 | | 34.5 | 2.03 | 79.6 |
|  | **RAR** | 1.03 | 1.29 | 25.2 | 1.24 | | 20.4 | 1.99 | 93.2 |

**Table 4**

Comparison of facet joint stress (MPa) in ACJC, CDA, ACCF, and intact model.

|  |  | **Intact** | **ACJC** | | **CDA** | | **ACCF** | |
| --- | --- | --- | --- | --- | --- | --- | --- | --- |
|  |  | **Von Mises stress (Mpa)** | **Von Mises stress (Mpa)** | **Increment (%)** | **Von Mises stress (Mpa)** | **Increment (%)** | **Von Mises stress (Mpa)** | **Increment (%)** |
| **C3-C4** | **FL** | 1.55 | 2.06 | 32.9 | 1.80 | 16.1 | 2.25 | 45.2 |
|  | **EX** | 1.69 | 2.16 | 27.8 | 1.91 | 13.0 | 2.23 | 32.0 |
|  | **LB** | 1.09 | 1.33 | 22.0 | 1.18 | 8.3 | 1.41 | 29.4 |
|  | **RB** | 1.21 | 1.47 | 21.5 | 1.32 | 9.1 | 1.57 | 29.8 |
|  | **LAR** | 2.05 | 2.45 | 19.5 | 2.17 | 5.9 | 2.62 | 27.8 |
|  | **RAR** | 1.87 | 2.22 | 18.7 | 2.01 | 7.5 | 2.43 | 29.9 |
| **C4-C5** | **FL** | 1.35 | 1.15 | -14.8 | 2.21 | 63.7 | 0.68 | -49.6 |
|  | **EX** | 1.49 | 1.32 | -11.4 | 2.23 | 49.7 | 0.94 | -36.9 |
|  | **LB** | 1.12 | 1.00 | -10.7 | 1.54 | 37.5 | 0.76 | -32.1 |
|  | **RB** | 1.04 | 0.94 | -9.6 | 1.45 | 39.4 | 0.78 | -25.0 |
|  | **LAR** | 1.87 | 1.63 | -12.8 | 2.36 | 26.2 | 1.32 | -29.4 |
|  | **RAR** | 1.74 | 1.50 | -13.8 | 2.18 | 25.3 | 1.29 | -25.9 |
| **C5-C6** | **FL** | 1.22 | 1.03 | -15.6 | 1.93 | 58.2 | 0.68 | -44.3 |
|  | **EX** | 1.37 | 1.15 | -16.1 | 1.95 | 42.3 | 0.85 | -38.0 |
|  | **LB** | 0.98 | 0.85 | -13.3 | 1.38 | 40.8 | 0.66 | -32.7 |
|  | **RB** | 0.84 | 0.72 | -14.3 | 1.19 | 41.7 | 0.58 | -31.0 |
|  | **LAR** | 1.83 | 1.55 | -15.3 | 2.21 | 20.8 | 1.19 | -35.0 |
|  | **RAR** | 1.84 | 1.52 | -17.4 | 2.18 | 18.5 | 1.17 | -36.4 |
| **C6-C7** | **FL** | 1.22 | 1.48 | 21.3 | 1.32 | 8.2 | 1.55 | 27.0 |
|  | **EX** | 1.32 | 1.56 | 18.2 | 1.47 | 11.4 | 1.63 | 23.5 |
|  | **LB** | 0.95 | 1.12 | 17.9 | 1.05 | 10.5 | 1.18 | 24.2 |
|  | **RB** | 0.91 | 1.07 | 17.6 | 0.99 | 8.8 | 1.14 | 25.3 |
|  | **LAR** | 2.20 | 2.49 | 13.2 | 2.35 | 6.8 | 2.59 | 17.7 |
|  | **RAR** | 2.01 | 2.31 | 14.9 | 2.19 | 9.0 | 2.41 | 19.9 |

**Table 5**

Comparison of maximum von Mises stress (MPa) in the cervical vertebra and the instruments in ACJC, CDA, and ACCF models.

|  | maximum von Mises stress of the cervical vertebra (MPa) | | | | maximum von Mises stress of the prostheses (MPa) | | |
| --- | --- | --- | --- | --- | --- | --- | --- |
|  | **ACJC** | **CDA** | **ACCF** | **ACJC** | | **ACDP** | **TMC-Plate-Screws** |
| **FL** | 10.3 | 43.1 | 11.0 | 116.7 | | 131.7 | 32.6 |
| **EX** | 16.7 | 50.6 | 26.8 | 160.9 | | 172.7 | 60.1 |
| **LB** | 35.5 | 57.8 | 24.1 | 129.1 | | 133.1 | 62.6 |
| **RB** | 28.2 | 52.6 | 24.6 | 124.0 | | 132.1 | 41.6 |
| **LAR** | 19.2 | 32.8 | 20.3 | 142.7 | | 142.4 | 43.4 |
| **RAR** | 18.4 | 36.4 | 19.7 | 144.7 | | 142.0 | 41.1 |
